# The broiler meat system in Nairobi, Kenya: Using a value chain framework to understand animal and product flows, governance and sanitary risks

**DOI:** 10.1016/j.prevetmed.2017.08.013

**Published:** 2017-11-01

**Authors:** Maud Carron, Pablo Alarcon, Maurice Karani, Patrick Muinde, James Akoko, Joshua Onono, Eric M. Fèvre, Barbara Häsler, Jonathan Rushton

**Affiliations:** a Royal Veterinary College (RVC), Hawkshead Lane, Hatfield, AL9 7TA, United Kingdom; b Leverhulme Centre for Integrative Research on Agriculture and Health (LCIRAH), 36 Gordon Square, London, WC1H 0PD, United Kingdom; c International Livestock Research Institute (ILRI), P.O. Box 30709, Nairobi, 00100, Kenya; d Institute of Infection and Global Health, University of Liverpool, Leahurst Campus, Chester High Road, Neston, CH64 7TE, United Kingdom; e University of Nairobi, P.O. Box 29053-00625, Nairobi, Kenya

**Keywords:** Chicken, Food system, Nairobi, Value chain, Governance, Disease risks

## Abstract

Livestock food systems play key subsistence and income generation roles in low to middle income countries and are important networks for zoonotic disease transmission. The aim of this study was to use a value chain framework to characterize the broiler chicken meat system of Nairobi, its governance and sanitary risks.

A total of 4 focus groups and 8 key informant interviews were used to collect cross-sectional data from: small-scale broiler farmers in selected Nairobi peri-urban and informal settlement areas; medium to large integrated broiler production companies; traders and meat inspectors in live chicken and chicken meat markets in Nairobi. Qualitative data were collected on types of people operating in the system, their interactions, sanitary measures in place, sourcing and selling of broiler chickens and products. Framework analysis was used to identify governance themes and risky sanitary practices present in the system.

One large company was identified to supply 60% of Nairobi’s day-old chicks to farmers, mainly through agrovet shops. Broiler meat products from integrated companies were sold in high-end retailers whereas their low value products were channelled through independent traders to consumers in informal settlements. Peri-urban small-scale farmers reported to slaughter the broilers on the farm and to sell carcasses to retailers (hotels and butcheries mainly) through brokers (80%), while farmers in the informal settlement reported to sell their broilers live to retailers (butcheries, hotels and hawkers mainly) directly. Broiler heads and legs were sold in informal settlements via roadside vendors.

Sanitary risks identified were related to lack of biosecurity, cold chain and access to water, poor hygiene practices, lack of inspection at farm slaughter and limited health inspection in markets.  Large companies dominated the governance of the broiler system through the control of day-old chick production. Overall government control was described as relatively weak leading to minimal official regulatory enforcement. Large companies and brokers were identified as dominant groups in market information dissemination and price setting. Lack of farmer association was found to be system-wide and to limit market access. Other system barriers included lack of space and expertise, leading to poor infrastructure and limited ability to implement effective hygienic measures.

This study highlights significant structural differences between different broiler chains and inequalities in product quality and market access across the system. It provides a foundation for food safety assessments, disease control programmes and informs policy-making for the inclusive growth of this fast-evolving sector.

## 1 Introduction

Livestock shape daily lives, by the provision of food and the use of scarce resources with potentially the greatest impacts in low to middle income countries which rely heavily on livestock for both subsistence and market sales. In Kenya, agriculture contributes 26% of its gross domestic product (GDP), and poultry represent roughly a third (30%) of the agricultural GDP (FAO, 2008). Populations of broiler and indigenous chicken have increased between 2006 and 2009 in Kenya and their proportions have also changed. In 2009, there were four times more indigenous chickens than broilers in the country, and 1.4 times more in Nairobi. In 2012, the reverse situation was observed with 1.6 times more broilers (409,715) than indigenous chickens (261,773) in Nairobi (GoK, 2012a). This was linked to a small decline in totals of indigenous birds and doubling of broiler birds between 2009 and 2012 (FAO, 2008). It illustrates the increasing concentration of commercial chicken farming in and around urban centres (e.g. Nairobi, Mombasa, Nakuru, Kisumu and Nyeri) where market access is guaranteed, compared to rural areas where indigenous chickens continue to dominate (Omiti and Okuthe, 2008). Nairobi, unlike other cities, has been found to be the final destination for poultry from across the country, and the major entry and transit point for poultry within the region (McCarron et al., 2015).

Consumption of poultry meat in Kenya is predicted to increase from 54.8 thousand metric tonnes in 2000 to 164.6 in 2030, and from 6 to 30.5 thousand metric tonnes in Nairobi (Robinson and Pozzi, 2011), due to urbanisation, population growth, economic growth making people wealthier, and the continuing viability of current broiler chicken systems (FAO, 2011b). To meet this expected demand growth, poultry production in Kenya is expected to increase from 56.9 to 1,666 metric tonnes by 2030 (FAO, 2008), again under the assumption that trade regulations remain similar and relative prices of inputs and outputs to the poultry system remain unchanged.

Since the 1960s Nairobi has experienced a rapid human population growth going from a city of 350,000 in 1962 to 3,375,000 in 2009. Many of these people are housed in the informal settlements which have increased with little official planning process (APHRC, 2014). Other changes have included more investment in better quality housing along with changes in shops and restaurants to satisfy the needs of a growing middle-class (AfDB, 2011, Kimenyi et al., 2016, Neven et al., 2009). These changes at both ends of the socio-economic spectrum are changing the use of land in and around the city and reducing the land area available for farming (Thuo, 2013). In the poultry sector the high level data indicate a change from indigenous breed chicken meat production which is partly reliant on scavenge based feed to an intensive broiler production systems with the need for concentrate feed and sophisticated systems of poultry breeding, in line with global trends (Narrod et al., 2008). The size of the units has increased and in some parts of the sector the scale of processing has gone towards industrial level slaughter and processing. While indigenous chicken rearing remains culturally important, commercial broiler chicken farming represents a production process with current input price levels that can supply affordable and accessible animal proteins, albeit a different product from the indigenous chicken (Omiti and Okuthe, 2008). It is argued that broiler chicken production is also better able to be incorporated into an urban environment, where land use pressures are increasing the average cost of land (FAO, 2011b). Where the birds are raised is on relatively small areas, the grain and oilseeds generated from more distant and less expensive land. The speed of growth of the birds also means that as many as six cycles of production can be completed in a year and therefore the profitability from these small areas of expensive land can compete with alternative uses.

Our understanding of what appears to be a logical progression of the broiler meat system is however limited to high level summaries. Value chain analyses are a powerful tool for understanding livestock production systems, their constituting chains and possible risk areas for disease spread within a sector. They comprise 1) the mapping and description of the value chains (i.e. identification of people involved in the production-supply continuum and routes to market livestock and their products), and 2) the characterisation of their governance (i.e. power dynamics, enforcement mechanisms, and institutional environment) (Rushton, 2009). There are a small number of value chain studies of the poultry sector in Kenya. Okello et al. (2010) investigated poultry value chains at district-level, the main study area being rural, but including some urban centres such as Nakuru. Its focus was to provide information for response planning for Highly Pathogenic Avian Influenza outbreaks. Kariuki et al.(2013) used a value chain approach for sampling meat bacterial zoonotic pathogens in Kenya that showed high levels of contamination in these chains. McCarron et al. (2015) involved a cross-sectional survey of backyard farmers, middlemen and traders in five of the eight Kenyan provinces.

To the authors’ knowledge, no study has been published to date investigating the exact market structure and linkages between people involved in the broiler chicken meat system with focus on Nairobi. Such information is essential to allow better planning for this sector, identification of growth opportunities, market development challenges and to support national food safety policies and disease control programmes. Given the challenges the sector faces in terms of food borne diseases (*Salmonella*, *Campylobacter*) (Meakins et al., 2003, WHO and FAO, 2009, Zhao et al., 2001), emerging disease issues such as avian influenza (Greger, 2007), and the ongoing intense debates on antimicrobial use in the intensive livestock systems with chicken specifically being focussed (Landers et al., 2012, Marshall and Levy, 2011, Van Boeckel et al., 2015), there is a need to assist broiler farmers in developing sustainable livelihood options and to identify food safety and health risks arising from these fast-evolving environments. It is therefore important to understand better the structure of this broiler meat system across income areas.

The aim of the study was to gain a detailed understanding of the structure, dynamics, sanitary risks and governance of the broiler chicken meat system of Nairobi using a value chain framework. The identification of drivers behind product flows and determinants of the system’s sanitary environment represents an important foundation for further governance assessment, food safety analysis and nutritional studies, of relevance for policy-making.

## 2 Material and methods

### 2.1 General overview

A cross-sectional study of Nairobi’s broiler meat value chains was implemented between February 2013 and April 2014, as part of a broader livestock value chains study. Data collection was qualitative and consisted of focus group discussions (FGD) and key informant interviews (KII). The key components of the broiler meat system studied included small-scale broiler farms, medium to large integrated broiler companies and their corresponding retailing channels (or “chains”), as well as the main live broiler and broiler meat markets in Nairobi. Research questions for the mapping objective (O1) were: what is the structure of the farm/market chains, from input sourcing to selling of outputs? and who are the people involved in the chains? Research questions for Objectives 2 and 3 (O2 and O3) were: what is the chains’ governance environment? and which practices present in the chains could affect sanitary risks?

### 2.2 Study area and selection of participants

Livestock production officers and veterinary officers from the Ministry of Livestock Development, Department of Veterinary services, were consulted to organise a series of broiler farmer focus group discussions and key informant interviews (Table 1). To encourage a diverse and representative pool of respondents, livestock officers were provided with guidelines to recruit the greatest possible diversity of farms and encouraged to go beyond their usual farm network. For data collection on small-scale broiler farms, two areas were purposely selected based on discussion with officials from the Ministry of Livestock Development, namely Dagoretti North and Kibera (Supplementary Fig. 1). Dagoretti North was selected due to its high livestock farming activity and peri-urban characteristics. A broiler population of 25,273 birds for Dagoretti North and South combined (Supplementary Fig. 1) is reported (GoK, 2012a). Kibera, the largest informal settlement in Nairobi, was selected to illustrate broiler farming in more densely populated and lower-income areas of the city. No broiler population data are available specifically for this area. Officers in charge of each area were asked to recruit a maximum of 12 broiler farmers from the area, as diverse as possible in terms of farm size, and to select local broiler brokers for FGD. For data collection on integrated broiler companies, interviews with key informants from two large companies and one medium company took place. Two Nairobi poultry markets were identified as important in terms of size, namely City market (the main broiler meat market in the central business district of Nairobi) and Burma-Maziwa market (the main live broiler market in the city). A series of interviews with each market’s meat inspector and a sample of market retailers took place as described in Table 1.

**Table 1:** Number of focus group discussions (FGD) and key informant interviews (KII) conducted in the study and characteristics of participants.

Data collection	Type/frequency	Type and number of participants, and size of flock owned, as applicable	Gender
Kibera small-scale farmers	1 FGD	9 Broiler farmers:	4M, 5F
		2 had > 100 birds	
		2 had 50–100 birds	
		5 had 15–50 birds	
Dagoretti small-scale farmers	2 FGD	9 Broiler farmers:	5M, 4F
		2 had > 1000 birds	
		3 had 301–1000 birds	
		4 had < 300 birds	
		9 Broiler brokers	5M, 4F
Integrated broiler companies (3)	3 KII	2 Government Veterinary officers onsite	3M, 1F
		2 Company managers	
City Market	4 KII	2 retailers (corridor vendor, Chairman of broiler retailers)	4M
		1 Meat Inspector,	
		1 Head of City Council	
Burma-Maziwa Market	1 FGD	5 Poultry traders	5M
	1 KII	1 Poultry inspector	1M

*Notes:* M: male; F: female.

### 2.3 Data collection

During small-scale farmers’ FGD, farmers were asked to identify and describe: 1) Flock size, types/sources of inputs (feed, water, day-old chicks (DOC)) used on the farm, indicative proportions for each input stream (O1); 2) Slaughtering/selling/transport processes, as applicable (O1); 3) Types of farm outputs (birds, meat, by-products), corresponding types of buyers/retailers, indicative proportions for each output stream (O1); 4) Challenges of buying and selling broilers and their products and barriers to entry (O2); 5) Rules for operating with other people (e.g. rules to follow to sell to different retailers) and views on dominance in the chains (O2); 6) Animal health management and waste disposal (O2).

Discussions were conducted in Kiswahili by bilingual Kenyan research members. Open-ended questions (e.g. what are the types of inputs used on the farm?) were used to investigate the six themes above, as well as prompts to explore further the diversity of activities, stakeholders and their interactions in the chains. Using a flipchart, the facilitators created jointly with the participants flowcharts describing the flows of people and products in the chains, and when possible the relative sizes of the flows. Flowcharts were amended until a consensus was reached. In addition to manual notes taken in English, discussions were video and audio-recorded and flipcharts retained. Interviews with key informants from medium to large integrated broiler companies also used open-ended questions regarding the six themes listed above, with additional questions regarding business characteristics (business structure/integration of activities). Facilitators recorded notes manually in English.

Markets data collection involved a mix of FGD and KII, which took place separately. A similar process was used to capture chain structure (buying and selling), governance and sanitary measures (themes 2–6 listed above). A series of predefined open-ended questions concerning the interviewee’s role in the market, sources and buyers of meat/birds, power-groups or rules in place, challenges to business, waste management and food safety risks, were used. In addition, researchers visited the markets and recorded their observations in terms of practices potentially risky for animal health/public health or food safety.

### 2.4 Ethical approvals and participant consent

Prior to data collection, ethical approvals were sought from the ILRI-IREC (International Livestock Research Institute – Institutional Ethical Research Committee, project reference ILRI-IREC2014-04/1). ILRI-IREC is accredited by the National Commission for Science, Technology and Innovation (NACOSTI) in Kenya. Approval from the Royal Veterinary College (RVC) ethical committee was also received (project reference: URN 2013 0084H). Permission to interview farmers was obtained from the Ministry of Agriculture and the local Veterinary Authorities. Prior to each FGD, the study’s objectives and participants’ rights were explained in Kiswahili to farmers and informants. Verbal and written consent to participate in the study were obtained before initiating discussions.

### 2.5 Data handling and analysis

The first data analysis step was to transcribe notes from each FGD and KII into a separate Word document template which followed the six broadly pre-defined thematic questions listed under the “Data collection” section. Through careful listening to audio recordings and review of qualitative data on flipcharts, data not already captured in the notes were added to the relevant sections of the template. This first step allowed structuring of the qualitative information gathered.

Subsequently, the mapping part of the study (O1) involved the creation of profiles (i.e. diagram representing people, flows of animals and products and other chain characteristics) for the key components of the broiler meat system: 1) Kibera small-scale farmers; 2) Dagoretti small-scale farmers; 3) medium and 4) large integrated broiler companies; 5) City market (meat market); and 6) Burma-Maziwa market (live bird market). For each profile, relevant data from FGD, interview templates and draft flowcharts were analysed and combined to create a detailed profile map. The main nodes in the chains (in terms of categories of farms, product sources and buyers) were identified and linked graphically by arrows to represent flows of people, animals and products. When possible, proportional size of flows was illustrated using arrows of different sizes. A brief description (as applicable) of flock size, inputs and outputs, was included in the graphical representation. Other data regarding interactions present within the chains was kept for the narrative explaining the profile.

For the second objective of the study (O2), a framework analysis was used to identify key determinants of governance and potential sanitary risk practices associated to each profile. The definition of governance included the type of rules in the system, sanctions and incentives, but also the nature of linkages between actors in the chains (based on Kaplinsky and Morris, 2000, Neven, 2014). Based on the broad topics used for FGD and KII and a first review of the templates, key categories of governance determinants and practices which could affect sanitary risks (“sanitary risk practices”) were identified. Governance determinants to be analysed included: 1) Dominant groups, including in terms of market information and technical knowledge; 2) Rules and incentives; 3) Challenges and business barriers, and 4) Farmer and trader associations. Sanitary risk practices of interest included: 1) Animal health services and practices; 2) Slaughter practices; 3) Farming/market/transport hygiene and biosecurity measures, and 4) Disposal of dead, condemned birds and by-products. Specific themes or practices for each category (e.g. “lack of capital” as an example of challenge, and “meat inspection” as a hygiene measure), were subsequently identified and coded by the main author. All categories and themes were reviewed by main co-authors to ensure proper categorisation and avoid gaps in theme identification. Findings are detailed by profile in a narrative in the result section.

## 3 Results

### 3.1 Structural components and flows

#### 3.1.1 Mapping of medium and large integrated broiler companies’ chains

**Fig. 1:**
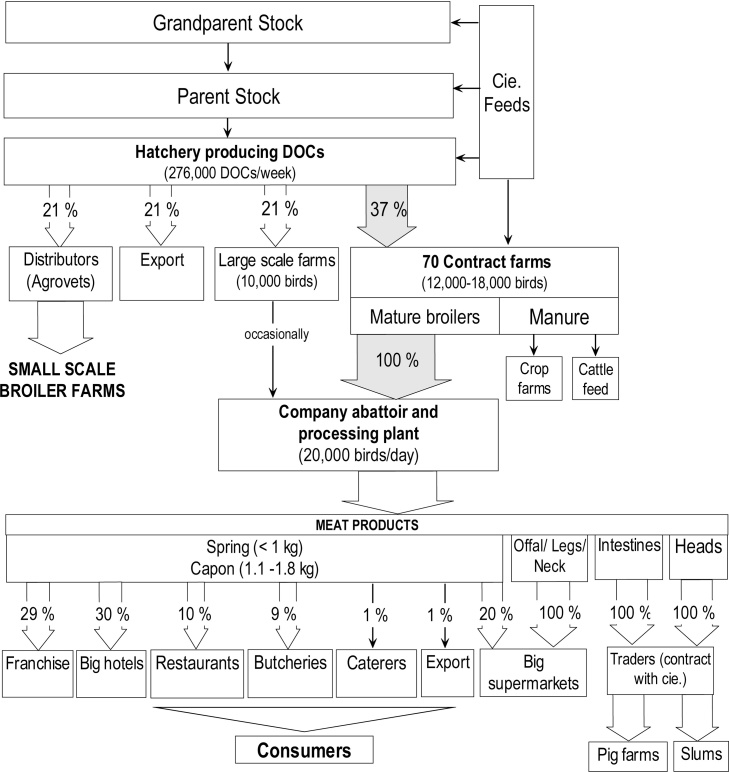
Large integrated broiler company (Cie.) profile – The flowchart indicates sources and flows of chickens/chicken meat in a nearly fully integrated production system (feed mill, grandparent stock, parent stock farms, hatchery and broiler abattoir are company-owned; broiler grower farms are contracted out). Notes: Large supermarkets include Nakumatt, Uchumi, and Tuskys. Carcasses (spring/capon) are exported to Tanzania, Uganda, Democratic Republic of Congo, Rwanda and Ethiopia.

Due to similarities between the large integrated broiler companies and medium-size integrated companies’ profiles, only the large companies’ profile is presented in detail (Fig. 1), with a mention of key profiles’ differences.

Genetic selection for grandparent and parent stock farms of the main large broiler company interviewed was reported to be done locally with no import of grandparents from Europe. Their hatchery was estimated by the informant to produce 60% of Nairobi’s DOC supply. The chicks were sold either to farms contracted by the company for fattening, exported to Uganda and Tanzania, sold to independent large scale broilers farms, or to agrovets (retailers of agricultural inputs including veterinary medicines) (GoK, 2012b). These agrovets are often owned and managed by personnel who have limited or no training in animal health (Higham et al., 2016).

The company had a contract with 70 broiler grower farms to raise the chicks to maturity. Mature broilers were bought back at 33–36 days of age to be slaughtered in the company’s abattoir. All contract farms were located within 100 km radius from the slaughter plant at the time of the interview. It was explained that in times of shortages the company would buy mature broilers from independent farms which had previously bought their DOCs from the company’s own hatchery.

The company-owned abattoir was situated on the outskirts of Nairobi; it produced 7.7 million kilograms of poultry meat in 2013. Nearly half of the DOCs produced by the company’s hatchery reached the abattoir for slaughter as mature broilers.

Meat and meat products were sold to high-end retailers across the city; intestines went to pig farmers as feed, and heads were bought by traders for resale in informal settlements. Processed products such as sausages, marinated chicken parts and burgers were reported to be produced only from layer birds, which were not included in this study.

Unlike the large company, the fully-integrated medium broiler company interviewed not only owned a hatchery (producing 20,000–40,000 DOCs/week), but also a broiler grower farm. Another key difference to the large company was the importation of parent stock (Cobb 500) from the UK (19,000 birds/year). The company’s hatchery supplied DOCs mainly to the company’s farm (60%) and to small-scale broiler farms in the city (30%). Export to countries in the region was lower than for the large company (10%). The company abattoir was reported to slaughter up to 10,000 birds/week, but on an irregular basis. Whole carcasses, special cuts and offals were sold mainly to restaurants (40%) and consumers at the abattoir-gate (40%).

#### 3.1.2 Mapping of small-scale broiler farm chains in Dagoretti and Kibera

**Fig. 2:**
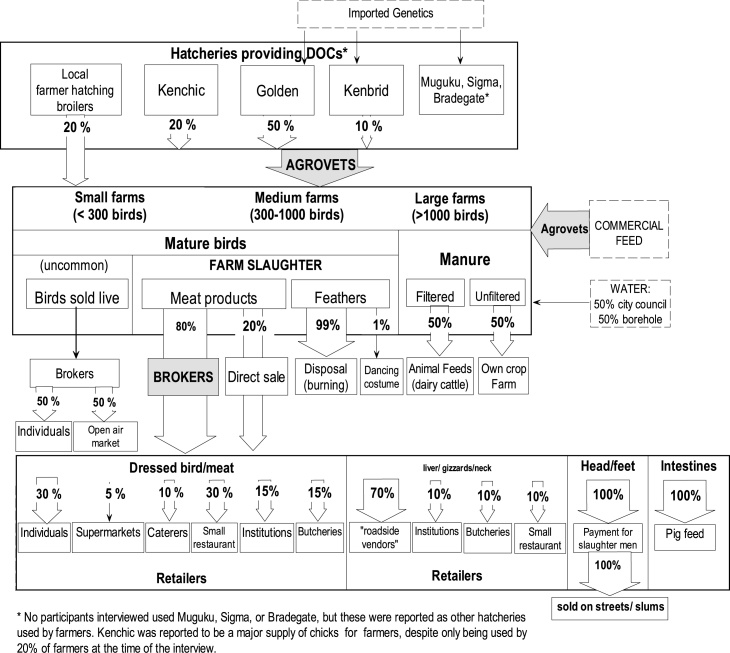
Dagoretti small-scale broiler farmers’ profile – The flowchart indicates sources of birds and retailing channels for chickens/chicken meat. Notes: Categories of farm size appear as defined by the focus group discussion participants. Roadside vendors: retailers selling products from a temporary stall in a specific street location.

**Fig. 3:**
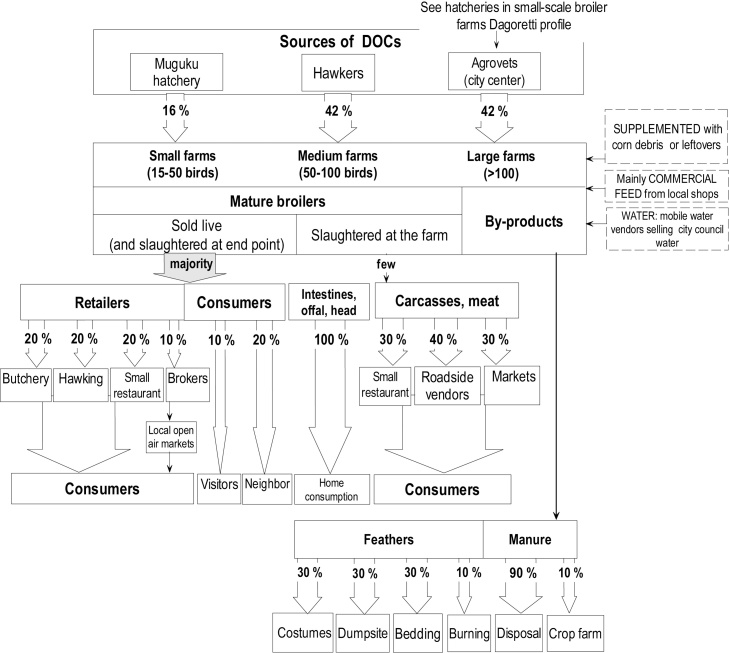
Kibera small-scale broiler farmers’ profile – The flowchart indicates sources of birds and retailing channels for chickens/chicken meat. Notes: Categories of farm size appear as defined by the focus group discussion participants. Roadside vendors: retailers selling products from a temporary stall in a specific street location.

The two profiles created for small-scale broiler farm chains in Dagoretti and in Kibera are presented in Fig. 2, Fig. 3, respectively; key differences are described here. Broiler grower farm sizes varied greatly between both areas. Most farmers interviewed in Dagoretti had flocks over 300 birds, whereas most participants in Kibera had under 100 broilers (Table 1). Dagoretti farmers reported that only six commercial hatcheries were being used for DOC supply to farmers via agrovets. Most hatcheries were said to import fertilised eggs from Europe as parent stock. In Kibera, only few farmers knew the hatchery of origin for their DOCs and otherwise reported using hawkers (retailers who move with their merchandise between markets) and agrovets as suppliers of young birds of unknown origin. Most farms in Dagoretti fed their broilers with processed commercial feed from two main feed companies using different feed preparations corresponding to the production phase. In Kibera, processed feed bought at local shops of unknown brand was used, mixed with house leftovers and human maize processing debris. Water used in Kibera was solely from city council water, either from public taps or water vendors, whereas in Dagoretti half of the farms used borehole water, and the other half city council water.

Most farms in Dagoretti used on-farm slaughter and sold their birds/meat products to brokers for further resale to retailers, whereas broilers in Kibera were mainly sold live, directly to a retailer. While chicken manure in Dagoretti was used on the farmer’s crops or sold as feed, it was mainly disposed-off in Kibera.

#### 3.1.3 Mapping of poultry markets’ chains

The Burma-Maziwa market (Supplementary Fig. 2) is composed of three “sub-markets”, the main section selling live indigenous chickens (Maziwa), and other sections selling a mix of live spent layers and broilers (Burma and mixed markets). Indigenous chickens were reported to originate from distant locations outside Nairobi (Kitui, Mwingi, Makueni, Bomet, Kisii, Kitale), whereas broilers and spent layers were supplied by farmers within the city and its surroundings (Ruai, Njiru, Huruma, Ruiru, Buru, Kiambu, Murang’a, Tigoni). Large integrated broiler companies were said to release their extended broilers in the market three times a week.

Participants of the traders’ FGD explained how brokers and traders were involved in supplying market retailers and individual buyers. The Burma part of the market was described to sell more broilers to retailers, and the Maziwa part more indigenous chickens to private consumers. Purchased birds were brought to the market’s poultry slaughter house where local staff slaughtered the bird and prepared the carcass. Heads, intestines and legs were bought mostly by women traders who would cook the parts and sell them in informal settlements inside Nairobi.

In the case of City market (Supplementary Fig. 3), the main meat market in Nairobi, the market’s meat inspector confirmed that slaughter took place at the farm of origin, and only indigenous chicken and broiler meat were sold on-site. Two types of retailers could be found in this indoor market: 1) permanent stall retailers and 2) corridor vendors, selling in temporary small stalls situated around the market’s indoor courtyard.

Half of the indigenous chicken meat was reported to be sourced from markets in Nairobi and half from independent retailers within the city, whereas broiler carcasses originated mainly from small-scale farmers (200–500 birds) located in Nairobi and its outskirts (Kiambu, Machakos, Mwingi, Nakuru, Ngong, Murang’a, Othaya, Embu). Some large broiler companies also sold frozen broiler products at the market. Brokers were only used in times of scarcity and difficult market access, while farmers used traders as an intermediary for the supply of meat to the market retailers. Meat market retailers in turn sold chicken meat to a variety of buyers.

### 3.2 Governance themes and practices which could affect sanitary risks

**Fig. 4:**
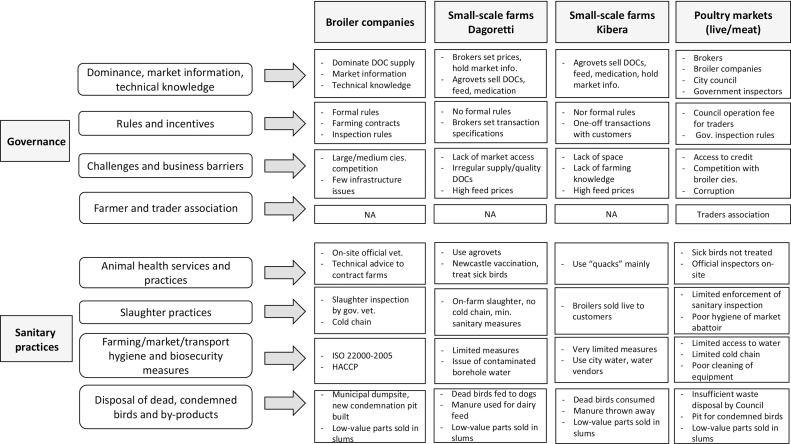
Analysis framework used for the identification of governance themes and sanitary risk practices present in the system. Notes: Info.: information; Gov.: government; Vet.: veterinarian; Min.: minimal.

Fig. 4 provides a graphic representation of the framework used and summary of key governance themes and practices which could affect sanitary risks; they are further explained in the narrative below.

#### 3.2.1 Governance themes

##### 3.2.1.1 Medium and large integrated broiler companies’ chains

Broiler companies were found to dominate the supply of DOCs in Dagoretti and Kibera through agrovets. They were identified as key knowledge-holders, providing technical expertise to small-scale broiler farmers, either as an incentive for engaging in contract-farming with large companies or for DOC purchase from their hatcheries. Formal rules in the large company’s operating environment were numerous. The large company’s contracts with broiler growers included provision of veterinary care and sanitary requirements, supportive of animal health and public health risk management. Unlike for small-scale farms, broiler companies were obliged to have a government veterinary officer overseeing their production. The latter was involved in issuing condemnation certificates, bird movement permits and meat export certificates. In terms of challenges, the medium company expressed difficulty in accessing the meat market due to competition with large companies. The large company mentioned a few infrastructure challenges, including lack of space/water access.

##### 3.2.1.2 Small-scale broiler farm chains in Dagoretti and Kibera

Broiler companies, brokers and agrovets were identified by small-scale farmers as dominant groups in the system, either in terms of market information dissemination, DOC supply or technical knowledge sharing. Kibera farmers described agrovets as the “go-to person”, not only for chick supply, but also for feeds, chicken medication and market information. Farmers sought technical advice regarding chicken farming from large companies in Dagoretti, and agrovets in both areas. Dagoretti farmers could not ensure continuous supply of chickens as demanded by retailers. They sold most of their chickens to brokers, who set prices and defined transaction modalities, unlike Kibera farmers who sold their chickens directly to retailers or consumers with no fixed transaction rules. No formal rules or interaction with the government services were reported by farmers. Only a few informal business rules (i.e. common processes found in the broiler business) such as farmer’s DOC ordering process from agrovets were described, as well as the use of discounts as purchase incentive.

Kibera farmers identified lack of space and farming knowledge, and high feed prices as their main business barriers. In Dagoretti, lack of market access, irregular supply and quality of chicks, and high feed prices were reported as key barriers. Lack of capital, and insufficient animal health and production trainings were also mentioned as impeding business development and farming quality. Finally, farmers linked the absence of farmer association in both areas to a lack of communication and trust between farmers.

##### 3.2.1.3 Poultry markets’ chains

In the case of the Burma-Maziwa and City markets, dominants groups not only included brokers and large broiler companies, but also the City council and government sanitary inspectors, responsible for ensuring hygienic practices and meat inspection. Multiple business and sanitary rules in place for market operations originated from those two last groups. In both markets, the City council required an operation fee from retailers and its responsibilities included waste collection and water supply. Requirements for health inspection by the government officers were in place in both markets (ante and post mortem inspection in Burma, and meat inspection in City market). Challenges included difficult access to credit, competition with large companies and police corruption. In both markets, traders reported the existence of a trader association responsible for championing their rights in relation to marketing and administrative matters (e.g. land-use rights, City Council responsibilities).

#### 3.2.2 Practices which could affect sanitary risks

##### 3.2.2.1 Medium and large integrated broiler companies’ chains

A high level of sanitary measures and controls was reported for the large broiler companies in comparison to small-scale farms. Biosecurity measures included ISO 22000-2005 certification (i.e. implementation of a food safety management system) of the slaughter plant, and presence of an HACCP (Hazard Analysis and Critical Control Points) system and a veterinary officer inspecting carcasses. Protective measures included sanitary requirements imposed by the large company in its contracts with grower farms. Details of the medium company’s hygiene processes were lacking, since the abattoir was not operating on the day of visit, but slaughter inspection by a government officer was mentioned as well as disposal of dead birds via burning. Some companies reported the ongoing building of a condemnation pit, to limit dependence from the municipal dumpsite, from which condemned birds had been retrieved by outsiders and sold illegally.

##### 3.2.2.2 Small-scale broiler farm chains in Dagoretti and Kibera

In case of disease, Dagoretti farmers rarely used veterinarians and only occasionally agrovets. Kibera farmers relied on services from “quacks”, i.e. community animal health worker with usually no formal training. Some protective practices were described, mainly in Dagoretti, such as Newcastle vaccination and treatment of sick birds before selling. Dagoretti farmers reported the use of on-farm slaughter for the broker, compared to live-selling in Kibera. Minimal sanitary measures (e.g. use of hot water) were described for such slaughter, with no cold chain available for the safe storage or transport of carcasses by the broker.

Farming hygiene and biosecurity measures were limited, especially in Kibera. A main concern of Dagoretti farmers in terms of water safety was the contamination of borehole water by pig waste and latrines. In Kibera, treated city tap water was used, but often sold by water vendors in dirty plastic barrels. Some practices of concern for zoonotic risks or inter-species disease transmission were reported. Dead birds were commonly consumed by farmers and their families in Kibera, whereas they were fed to dogs or pigs in Dagoretti. Chicken manure was used as dairy feed in Dagoretti and thrown away in the open sewage system in Kibera, and feathers were mainly burnt. In both areas, most low-value chicken parts, presenting the highest potential for faecal contamination, were sold to consumers in informal settlements.

##### 3.2.2.3 Poultry markets’ chains

The framework analysis of risky practices revealed a poor level of hygiene in the market environments and multiple biosecurity breaches. In both cases, sanitary inspection was required, but participants reported little inspection enforcement, due to poor resource allocation. Inspectors in both markets linked rule-breaking by traders, and lack of implementation of hygiene measures by retailers, to a lack of knowledge. In Burma market, sick animals were not treated. It was reported that small restaurants often bought animals which had died. Burma’s abattoir infrastructure and hygiene was reported and observed to be poor, with stone surfaces difficult to clean and no running water. In both markets, permanent identification (i.e. stamping) of inspected meat did not occur, making the identification of safe meat difficult. City market chicken meat was mainly kept at ambient temperature during the day and stored overnight in freezers, when not sold. Lack of water for cleaning was a major issue in both markets. While Burma had no running water (only water tanks), City market had some running water, but in insufficient quantity (it ran out early every day). This translated in lack of cleaning of meat cutting equipment and the working environment, increasing the risk of cross-contamination. Corridor vendors in City market were reported to commonly wash chicken carcasses in water buckets (not readily cleaned) before wrapping them in plastic bags, a practice potentially contributing to bacteria proliferation. There was no reference to dedicated transport means for birds or meat, rather crates and public buses were used. Finally, Burma had no toilets and City market only a paying-one, which increased the risk of workers contaminating carcasses with unwashed hands. Sewage was present in both systems. In City market, the positioning of corridor vendors above the open drainage lines favoured contamination of their products. A lack of waste collection by the City council was a common issue cited by participants, leading to garbage accumulation, rotting of waste and an unhygienic environment. One participant attributed this low level of service to insufficient City council employees. Burma had a dumping pit for condemned birds, some of which were reported to “found their way to hotels (small restaurants)”.

## 4 Discussion

The study provides a detailed characterisation of broiler chicken and broiler chicken meat flows in Nairobi, including persons involved in the farming and retailing of these products, and enabled identifying governance themes and potentially risky practices in the meat system. This combined approach, linking value chain mapping and framework analysis of the broader sanitary environment, is in-line with the Food and Agriculture Organization’s (FAO) recommendation to promote value chain analysis in animal diseases risk management (FAO, 2011a). The level of detail achieved in the system mapping is unique for the Nairobi broiler meat system, and allows a thorough understanding of its structure. It complements the Okello et al. (2010) study, one of the few other analyses detailing Kenyan poultry value chains in districts outside Nairobi.

The mapping of broiler farms and markets identified key differences in broiler production types, chain structure and product marketing. Chain structure varied in terms of length and complexity between profiles. Short, simple chains were found in Kibera small farms profile, whereas chains in Dagoretti involved more intermediaries, such as brokers. Longer chains have been linked to increased transaction costs in the system, often to the benefit of traders, but to the detriment of farmers (Okello et al., 2010, Shiferaw et al., 2008). Despite these transaction costs, African farmers commonly engage in selling at the farm-gate to brokers or other buyers to access cash quickly or limit transport cost (Fafchamps and Vargas Hill, 2005). In both study areas, small farm chains presented great variability and diversity in their retailing channels. The framework analysis echoed these findings, describing selling transactions in Kibera as “one-off” and driven by personal considerations such as acquaintance with a buyer, and transactions in Dagoretti as dictated by broker’s decisions, with no formal contracts between brokers and farmers. Lack of market knowledge was a recurring challenge cited by small-scale farmers, which could partly explain the volatility of the chains. This irregularity of transactions and lack of stable market access in the chicken meat system diminishes the attractiveness of the business for many small-scale farmers (Okello et al., 2010). Large-scale companies’ chains on the other hand were found to be more structured. Their key role in Nairobi’s broiler industry, influence on prices, and the use of formal contracts with broiler growers, lead to more stable chains.

Supply of DOCs in all profiles was dominated by a few large companies’ hatcheries, and in particular the DOC production of one large integrated company, with similar high concentration of broiler slaughter through integrated abattoirs. The importance of this flow in the meat system, which could be described as a monopoly, was confirmed by the framework analysis, in which farmers and market retailers identified large companies as dominant groups, and their competition as a main challenge. This dominance in terms of DOCs supply has been found to apply to the whole of Kenya, not only to Nairobi (Okello et al., 2010), and reflects the current global structure of the internationally integrated poultry industry (Manning and Baines, 2004, Narrod et al., 2008). The limited number of commercial hatcheries and their business structure (i.e. integrated production, and formal grower farms contracts) translate into a stable supply of DOCs of homogenous genetics and high quality across the system, while also making the system fragile to market shocks and pandemics (Omiti and Okuthe, 2008). This dominance could have wide-reaching implications, should a policy change occur. A loss of market for the company or reduction in productivity due to a disease outbreak could have food security repercussions or drastically change the system’s dynamics in terms of demand and market access for smaller producers. Other major flows (in terms of relative volume) identified in the mapping included flows of broiler meat sold by brokers in Dagoretti, and supply of feed and DOCs by agrovets in both areas. These flows once more corresponded to dominant groups, namely brokers holding market information, and agrovets holding technical knowledge. These parallel findings between the mapping and framework analysis indicate complementary approaches, enabling a more detailed understanding of the system.

The lower value parts of chicken carcasses (heads, feet), whether coming from Dagoretti farms, large/medium companies’ abattoirs, or Burma/City Markets’ retailers, were consistently directed to consumers in informal settlements via traders. This illustrates a well-established informal market structure, harbouring distinct channels for consumers of different socio-economic status. It raises the issue of the nutritional value and food safety of these lower-value products, as a main chicken meat input in informal settlements (Cornelsen et al., 2016). Another example of dual market structure in Kenya is the milk market, where informal channels dominate despite an important commercial milk sector. In the latter case, however, milk products of the formal sector have been shown to present similar health risks to the ones from other channels (IIED, 2015). Trade-offs between the food-security potentials of chicken meat in low-income areas and improvement of food safety (IIED, 2015, Cornelsen et al., 2016) will have to be evaluated in future research.

In terms of barriers to production, farm size was found to be limited in the densely populated Kibera environment, compared to the peri-urban Dagoretti area. Framework analysis revealed that farmers considered space as a main challenge for production, illustrating the effect of the urban environment on production practices. This questions the feasibility of commercial farming in densely populated areas, where small-scale farming is already a challenge due to a context of unsecured land occupation (Martellozzo et al., 2014, FAO, 2011b). Other challenges identified included lack of capital, technical knowledge and equipment. These could explain the poor farming infrastructure and biosecurity practices found in both areas, and to a greater extent in Kibera. Participants in Kibera reported consumption of dead birds, whereas in Dagoretti these were given to pigs/dogs, indicating also differences in access to health information and socio-economic status.

The identification of rules and other governance themes helped to understand further some of the drivers behind risky practices present in the system. The lack of rules found in the small-scale farms profiles, echoed their chains’ variability and informal structure. Very little animal health and biosecurity practices were in place in small farms. On the contrary, sanitary risk management was best addressed by the large companies’ set of sanitary rules and inspection processes (HACPP and ISO systems, production standards through farming contracts). Other studies demonstrated the parallel between a higher level of governance structure or formal arrangements, and enhanced biosecurity (Okello et al., 2010, Rushton, 2010). This raises the concern of higher prices for safer food and questionable accessibility of quality products for the majority of the Nairobi’s population living in informal settlements (APHRC, 2014).

Despite representing a more controlled environment (e.g. inspection rules in place) in comparison to farms, markets showed very little hygiene and biosecurity measures overall. Waste disposal by the City council was irregular, inadequate access to water lead to unsafe or absent cleaning practices, increasing the risk of cross-contamination, and cold chain was lacking, increasing the perishability of the meat. The limited resources available for regulatory enforcement observed in markets, despite the potential for disease spread in these high throughput and high contact network nodes, is of concern. We hypothesize that markets’ benefits must outweigh their risks, or that markets’ disease burden is still poorly understood, for the situation to be maintained. Indeed, Nairobi is a major hub for poultry marketing (McCarron et al., 2015) and poultry markets not only represent a key source of income for traders and farmers, but also for the City. Through markets, access to chicken products is centralised, in a system where marketing links are not formalised (except in the case of large companies). It will be important to investigate further public health impacts of such key nodes. Food safety consequences may evolve with growing middle class, increasing urbanisation and changes in exposure to pathogens, all determinants of host immunity (Havelaar et al., 2009).

Lack of trust and communication were mentioned by farmers as reasons behind the lack of farmer association in both Dagoretti and Kibera, which illustrates how policies improving technical and market knowledge at small-farmer level could have a positive impact on business development. Examples of poultry farmers’ associations, in charge of poultry selling and price negotiation, have shown to reduce the marketing powers of brokers in other parts of Kenya (Okello et al., 2010). One of these bought birds from farmers for processing at the association’s slaughter plant, thus stabilising market demand for farmers and creating value-addition opportunities. This approach could be considered for Nairobi, where a significant gap is the lack of poultry abattoirs available to independent farmers. A legal framework supporting the creation of farmer cooperatives could also facilitate farmer’s access to credit (Shiferaw et al., 2008). Unlike farmers, traders in both markets studied had formed an association, in hope to improve their negotiation power with the Council. This initiative raises the question of government involvement versus private implementation of sanitary safeguards, which has the potential to translate into higher biosecurity (Rushton, 2010). In a setting where corruption issues have been reported by participants, and hinder the fulfilling of some official sanitary roles, the potential benefits of private initiatives for the industry call for further investigation.

The study presented some limitations, which need to be considered when interpreting the results. Data collected are qualitative in nature and based on FGD and KII. Findings are therefore based on perception and opinions which can translate into some approximations. Although it was impossible to interview a large sample of people due to time and resource constraints, key informants selected had great knowledge of the area and industry, complementing FGD to qualitatively present the situation as described by stakeholders and the relative size of flows. To avoid response bias, FGDs involved participants from a same broad group (e.g. only farmers, or only traders), and were held separately from KIIs. While there is nothing to indicate a lack of diversity in the group of farmers interviewed, potential selection bias in farmers’ recruitment may have occurred and led to the inclusion of farms with best practices. This “best case scenario” in terms of risk, would not significantly impact flows of products in the profiles, which were found to be varied. In contrast, interviewing farms with best practices would not allow capturing lower sanitary standards being applied in other farms. Considering the near-absence of biosecurity measures reported in the study, risk practices found in farms of lower sanitary status should not differ greatly, thus not impacting significantly the representativeness of the study. No validation by external experts was done, however data were triangulated to confirm consensus in the case of markets, where retailers and officers were interviewed, and for Dagoretti small-scale farms, where farmers and brokers described the same value chains. Another limitation of the study resides in its geographic scope, FGD covering only two areas of Nairobi. While Dagoretti and Kibera represent major areas, whether in terms of farming intensity or human population size, the results cannot be fully extrapolated to the whole city. However, in such a volatile and diversified environment, with omnipresent informal chains, data collected enabled understanding the system’s contrasts in terms of socio-economic settings and allowed identifying patterns, key interactions between people, and main flows of products.

The very detailed information provided by the study has many applications, for the Nairobi as well as the East African context. Due to ongoing urbanisation in East Africa, the understanding of broiler systems in informal settlements and peri-urban areas is relevant for urban planners who will face increasing numbers of farms within cities. It provides a framework for designing food safety studies’ sampling frames, which is an essential preliminary step for prevalence studies and can be applied to other geographic areas. Some risk hot spots identified in the system (e.g. markets’ waste management, broiler on-farm slaughter), whether in terms of throughput of animals/animal products or presence of risky practices, can guide the elaboration of disease control programmes. Dominant groups identified in the governance analysis can be considered as potential levers in the system and included in the design of intervention programmes. Agrovets, a key link in the farmers’ network, could be the focus of animal health training programmes. Some accreditation schemes, like the one from the Kenyan Dairy Board for small-scale milk vendors (IIED, 2015), may support vulnerable broiler farmers in joining formal chains and improve the system’s biosecurity.

## 5 Conclusions

The use of value chain mapping and framework analysis for understanding the structure of a fast-evolving system, as well as its risk practices and governance, presents novelty. This study highlights significant structural differences between different broiler chains. Inequalities in product quality and market access found across the system were significant. To thrive from the food safety and income generation potentials of the commercial chicken sector, Kenya will need inclusive policy-making for progressive small-holder involvement and formalisation of the chains. As a major Kenyan hub for poultry marketing, Nairobi has a key role to play in shaping the system’s approaches to growth. Future research should also pay attention to the growing middle-class consumers’ preferences in planning system changes.
