# Supplementary material for: The broiler meat system in Nairobi, Kenya: Using a value chain framework to understand animal and product flows, governance and sanitary risks
Source: Prev Vet Med. 2017 Nov 1;147:90–9. doi: 10.1016/j.prevetmed.2017.08.013 (PMC5744866; doi:10.1016/j.prevetmed.2017.08.013)
Supplement: Supplementary file 1 [file mmc1.docx]

Supplementary Figure 1: Close-up map of Nairobi, Kenya (left) - It indicates the location of small-scale broiler farmers, broiler integrated companies and broiler markets included in the study; Map of Kenya (right).
