# Supplementary material for: The broiler meat system in Nairobi, Kenya: Using a value chain framework to understand animal and product flows, governance and sanitary risks
Source: Prev Vet Med. 2017 Nov 1;147:90–9. doi: 10.1016/j.prevetmed.2017.08.013 (PMC5744866; doi:10.1016/j.prevetmed.2017.08.013)
Supplement: Supplementary file 2 [file mmc2.docx]

Supplementary Figure 2: Burma-Maziwa market profile – The flowchart indicates sources and flows of live chicken in the market. Note: The market has an on-site slaughter house.
