# Supplementary material for: The broiler meat system in Nairobi, Kenya: Using a value chain framework to understand animal and product flows, governance and sanitary risks
Source: Prev Vet Med. 2017 Nov 1;147:90–9. doi: 10.1016/j.prevetmed.2017.08.013 (PMC5744866; doi:10.1016/j.prevetmed.2017.08.013)
Supplement: Supplementary file 3 [file mmc3.docx]

Supplementary Figure 3: City market profile – The flowchart indicates sources and flows of chicken meat in the market. Notes: This is the main meat market in Nairobi. No chicken slaughter takes place on-site.
